# Antibacterial Activity of Chinese Red Propolis against *Staphylococcus aureus* and MRSA

**DOI:** 10.3390/molecules27051693

**Published:** 2022-03-04

**Authors:** Wenwen Zhang, Gomez Escalada Margarita, Di Wu, Wenqin Yuan, Sha Yan, Suzhen Qi, Xiaofeng Xue, Kai Wang, Liming Wu

**Affiliations:** 1 Institute of Apicultural Research, Chinese Academy of Agricultural Sciences, Beijing 100094, China; zhangwen_w@126.com (W.Z.); yanshawell@163.com (S.Y.); suzhen_qi@126.com (S.Q.); xue_xiaofeng@126.com (X.X.); 2 School of Health, Leeds Beckett University, Leeds LS1 3HE, UK; m.gomez-escalada@leedsbeckett.ac.uk; 3 College of Animal Science, Shanxi Agricultrual University, Jinzhong 030801, China; wu11092021@126.com; 4 School of Life Science, Liaocheng University, Liaocheng 252000, China; yuanwenqin2020@163.com

**Keywords:** Chinese red propolis, antibacterial activity, metabolomics, antibacterial mechanism

## Abstract

The antibacterial activity of propolis has long been of great interest, and the chemical composition of propolis is directly dependent on its source. We recently obtained a type of propolis from China with a red color. Firstly, the antibacterial properties of this unusual propolis were determined against *Staphylococcus aureus* and methicillin-resistant *Staphylococcus aureus* (MRSA). Studies on its composition identified and quantified 14 main polyphenols of Chinese red propolis extracts (RPE); quantification was carried out using liquid chromatography triple quadrupole tandem mass spectrometry (LC-QQQ-MS/MS) and RPE was found to be rich in pinobanksin, pinobanksin-3-acetate, and chrysin. In vitro investigations of its antibacterial activity revealed that its activity against *S. aureus* and MRSA is due to disruption of the cell wall and cell membrane, which then inhibits bacterial growth. Despite its similar antibacterial activities against *S. aureus* and MRSA, metabolomic analysis further revealed the effects of RPE on bacteria metabolism were different. The untargeted metabolomic results showed that a total of 7 metabolites in 12 metabolic pathways had significant changes (Fold change > 2, *p* < 0.05 *) after RPE treatment in *S. aureus*, while 11 metabolites in 9 metabolic pathways had significant changes (Fold change > 2, *p* < 0.05 *) after RPE treated on MRSA. Furthermore, RPE downregulated several specific genes related to bacterial biofilm formation, autolysis, cell wall synthesis, and bacterial virulence in MRSA. In conclusion, the data obtained indicate that RPE may be a promising therapeutic agent against *S. aureus* and MRSA.

## 1. Introduction

Propolis is a resinous substance collected by honeybees (*Apis mellifera*) when they collect natural plant shoots, resinous secretions, pollen, and soil and mix it with their own glandular secretions to repair the hive and protect it from external aggression [1]. The chemical composition of propolis is very complex and is directly related to the local plant source of the honeybees [2], mainly including polyphenols, flavonoids, terpenoids, quinones, minerals [3], etc. Depending on its botanical origin and color, propolis is classified as green, red, or brown in Brazil [4]. *Baccharis dracunculifolia* DC (Asteraceae) in southeastern Brazil is the main botanical source of green propolis [5]; red propolis is found in bee hives along the coast and mangroves of northeastern Brazil, and its botanical source is *Dalbergia ecastophyllum* (L.) Taub [6]. Most Chinese propolis is greenish-black or dark in color [7] and is typical of poplar propolis, whose main botanical source is poplar (*Populus* sp.) [8].

The biological activity of propolis is closely related to its chemical composition which in turn is linked to the botanical origin of the plants the honeybees visit [9]. Propolis has extensively reported wide ranging biological activity which in addition to antibacterial activity includes antioxidant, antitumor, immunomodulatory [10], and anti-inflammatory [11] activities.

The antibacterial activity of propolis has been of great interest to researchers, and the antibacterial activity of a variety of different propolis from all over the world has been studied. Couto et al. studied and demonstrated the in vitro inhibitory activity of Brazilian brown propolis against *Enterococcus faecalis* [12]. Silici and collegues studied the chemical composition and antibacterial activity of *A. mellifera* propolis from three different honeybee races. Forty-eight compounds were identified by GC/MS and the ethanolic extract of propolis was evaluated for antibacterial activity against *Staphylococcus aureus*, *Escherichia coli*, *Pseudomonas aeruginosa*, and *Candida albicans*. The results showed that propolis showed high antibacterial activity against Gram-positive bacteria and weaker activity against Gram-negative bacteria and yeasts [13]. Further studies on the antibacterial mechanism of action of propolis have postulated that propolis may reduce ATP production, increase membrane permeability, decrease bacterial motility, and/or disturb membrane potential [14].

*Staphylococcus aureus* (*S. aureus*) [15] and methicillin-resistant *Staphylococcus aureus* (MRSA) are two pathogens that pose a significant threat to public health worldwide [16]. *S. aureus* causes infections of the skin and soft tissues and with the extensive use of antibiotics, *S. aureus* is gradually acquiring mechanisms of resistance to various antibiotics [17], leading to the emergence of MRSA in 1961 [18]. The use of various antibiotics can also have negative effects on the body alongside their antibacterial action, via increasing the metabolic burden on the kidneys, inactivating surface activity in the lungs, and inhibiting bone marrow [19]. MRSA is of great scientific interest, with a recent study finding that an increased proportion of antibiotic resistance in MRSA was found in patients with significant pathogenicity and mortality [20]. Hence, there is an urgent need to find and develop natural antibacterial substances that can reduce the metabolic burden on the body whilst possessing antimicrobial effectiveness.

Metabolomics is the qualitative and quantitative study of small molecule metabolites (<1000–1500 Da), one of the main objectives of which is to identify biomarkers that have a direct impact on the metabolism or metabolic pathways of an organism [21]. Metabolomics applied to the study of antibacterial activity by analysis of changes in bacterial metabolites and metabolic pathways can reveal antibacterial mechanisms of action. Chen et al. used metabolomics to explore the antimicrobial mechanism of essential oil from the leaves of cinnamon tree (*Cinnamomum camphora* (Linn.) Presl using MRSA as a model, the results showed 74 differential metabolites—29 of which were upregulated and 45 downregulated—and a total of 7 pathways were enriched by common differential metabolites [22]. Non-targeted metabolomics was applied by He et al. to assess the mechanism of inhibition of *Lysteria monocytogenes* by linalool and found significant changes in metabolites involving amino acid, central carbon, lipid, and nucleic acid metabolisms [23].

**Figure 1:**
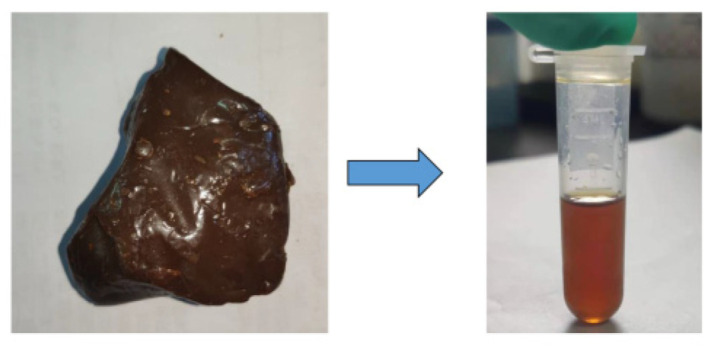
Chinese red propolis extracts from Shandong, China. Left is Chinese red propolis extract, right is Chinese red propolis extract dissolved in methanol.

Most reports on red propolis come from Cuba and northern Brazil [24]. In recent years, red propolis from China also has been discovered and investigated, researchers have reported that red propolis from China has strong antioxidant activity [25] and inhibits vascular endothelial growth factor-induced angiogenesis [26]. In this study, the main objective is to evaluate antibacterial activity of Chinese red propolis (Figure 1) against *Staphylococcus aureus* and MRSA and elucidate the mechanism of action. The effects of Chinese red propolis extracts (RPE) on the integrity of bacterial cell walls and cell membranes were observed, and the changes in metabolites after the action of RPE on *S. aureus* and methicillin-resistant *S. aureus* were also analyzed by LC-QTOF-MS/MS. We hope to better illustrate the inhibition mechanism of CRP on *S. aureus* and MRSA.

## 2. Results

### 2.1. Results of the Quantitative Analysis of Polyphenols in RPE

Quantification of the 14 polyphenols in RPE was achieved using Triple Quadrupole LC-MS/MS detection conditions for each of the 14 polyphenol standards MS/MS parameters were optimized with multiple reaction monitoring (MRM). The selected parameters including precursor, product ions, collision energy (CE), and quantification results for all polyphenols are listed in Table 1. MS/MS spectra of 14 polyphenols are shown in Supplementary Figure S1.

### 2.2. RPE Has Anti-Bacterial Effect on S. aureus and MRSA

In this study, we utilized two strains of *Staphylococcus aureus* ATCC 25923 which is standard bacterial strain, and MRSA ATCC43300 strain. RPE was shown to have significant anti-bacterial effects against both bacterial strains. The zone of inhibition of RPE against *S. aureus* and MRSA using an agar-well diffusion test as previously described were assessed, methanol was used as a solvent control. Based on previous studies, the zone of inhibition diameter was used to ascribe descriptors of insensitive (≤8.0 mm), moderately sensitive (8.0 < diameter < 14.0 mm), sensitive (14.0 < diameter < 20.0 mm), and extremely sensitive (≥20.0 mm) [27]. Our study showed the inhibition zone of RPE against *S. aureus* to be 16.5 ± 0.5 mm, and the zone of inhibition of RPE against MRSA to be 19.3 ± 0.5 mm. No inhibition zone was found in the control group. The MIC and MBC of RPE against *S. aureus* and MRSA were determined by the microbroth dilution assay and the results are shown in Table 2. An MIC of 100 µg/mL for *S. aureus* and an MIC of 50 µg/mL for MRSA was determined, whilst the MBC of RPE for *S. aureus* and MRSA were found to be 300 µg/mL and 200 µg/mL respectively.

### 2.3. Time–Growth Curve Analysis

**Figure 2:**
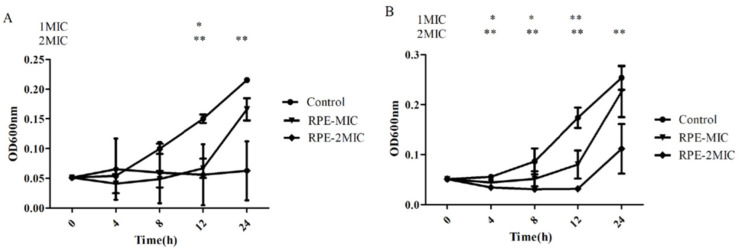
Time-killing curve of RPE against *S. aureus* (**A**) and MRSA (**B**). (**A**) The time growth curve of RPE against *S. aureus*. (**B**) The time growth curve of RPE against MRSA. All data are presented as mean values ± SD, and *n* = 3 in each group. * *p <* 0.05, ** *p <* 0.01, vs. the solvent control.

Following determination of the MIC and MBC of RPE against *S. aureus* and MRSA, we studied the growth curves of *S. aureus* and MRSA treated with MIC and 2 × MIC concentrations of RPE over 24 h, as shown in Figure 2. RPE showed significant inhibitory activities on *S. aureus* and MRSA in a concentration-dependent manner, the viability of *S. aureus* and MRSA were significantly (*p <* 0.05) inhibited by RPE in the 24 h following its administration.

### 2.4. Inhibitory Effect of RPE on Intracellular Protein and DNA Leakage Analysis

**Figure 3:**
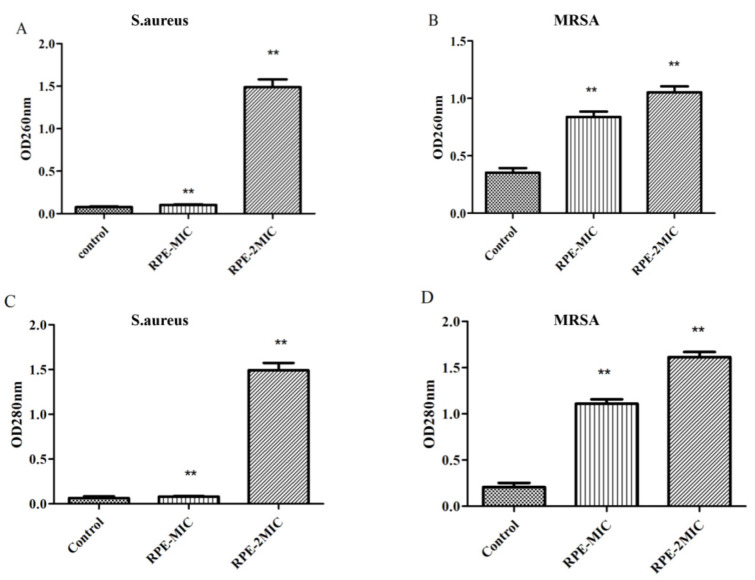
260 nm absorbance values of *S. aureus* (**A**) and MRSA (**B**) treated with RPE, and 280 nm absorbance from *S. aureus* (**C**) and MRSA (**D**) treated with RPE. All data are presented as mean values ± SD, and *n* = 3 in each group. ** *p* < 0.01, vs. the solvent control.

In this study, we further tested the effects of RPE treatment on the bacterial cell wall membrane integrity. *S. aureus* and MRSA cell wall membrane integrity were investigated by analyzing the absorbance of bacterial supernatant due to release of nucleic acid at 260 nm and protein at 280 nm. Our results on OD260 and OD280 assay (Figure 3), show RPE treatment (to concentrations of MIC and 2 × MIC) of *S. aureus* and MRSA significantly increased the OD260 and OD280 values (*p* < 0.05) in a concentration dependent manner, indicating that RPE can inhibit the synthesis of DNA and proteins in *S. aureus* and MRSA, and the integrity of the cell membrane is significantly impacted by RPE.

### 2.5. Cell Wall Integrity Analysis

**Figure 4:**
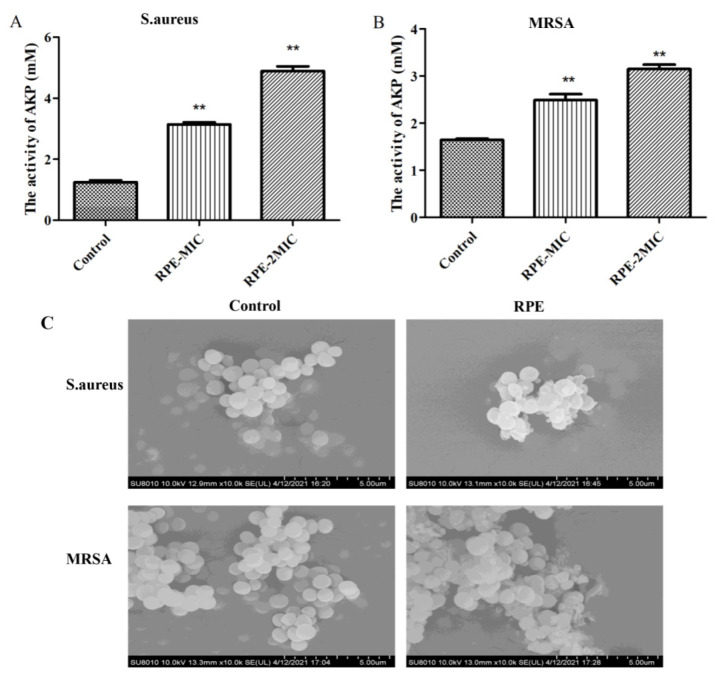
Assessment of leakage of AKP from (**A**) *S. aureus* and (**B**) MRSA treated with RPE; and (**C**) scanning electron micrographs of *S. aureus* and MRSA treated with RPE. Scanning electron micrographs show cell wall integrity of *S. aureus*: (**left**) *S. aureus* control (magnification × 100,000); (**right**) *S. aureus* treated with the RPE at 100 µg/mL (magnification × 100,000); Scanning electron micrographs showing cell wall integrity of MRSA: (**left**) MRSA control (magnification × 100,000); (**right**) MRSA treated with the RPE at 100 µg/mL (magnification × 100,000). All data are presented as mean values ± SD, and *n* = 3 in each group. ** *p* < 0.01, vs. the solvent control.

The effects of AKP leakage from bacteria are presented in Figure 4A and Figure 4B, compared to the control. The leakage of AKP from *S. aureus* (A) and MRSA (B) treated with RPE increased significantly in a dose-dependent manner. Figure 4C shows SEM images of *S. aureus* and MRSA treated with RPE alongside an untreated control. SEM images show cell wall damage in *S. aureus* and MRSA treated with 100 µg/mL of RPE. Untreated *S. aureus* and MRSA cells were spherical in shape, with a smooth surface and retained normal cell morphology. Following treatment with RPE, cells became irregular, exhibiting deformation of cell membranes in both *S. aureus* and MRSA groups.

### 2.6. Metabolomic Analysis of the Effect of RPE Treated on S. aureus and MRSA

**Figure 5:**
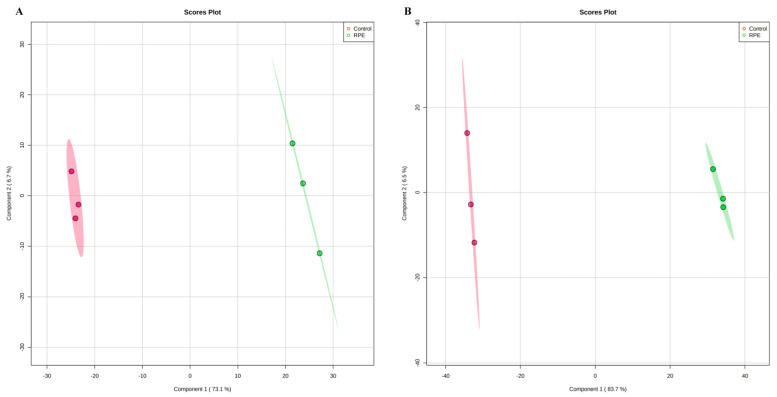
OPLS-DA spot of RPE treated on *S. aureus* and MRSA compared with untreated respectively. (**A**) OPLS-DA analysis of effects of RPE treatment of *S. aureus* relative to control. Each point represents a duplicate sample in each group (*n* = 3). (**B**) OPLS-D analysis of effects of RPE treatment of MRSA versus control. Each dot in the figure represents a duplicate sample in each group (*n* = 3).

**Figure 6:**
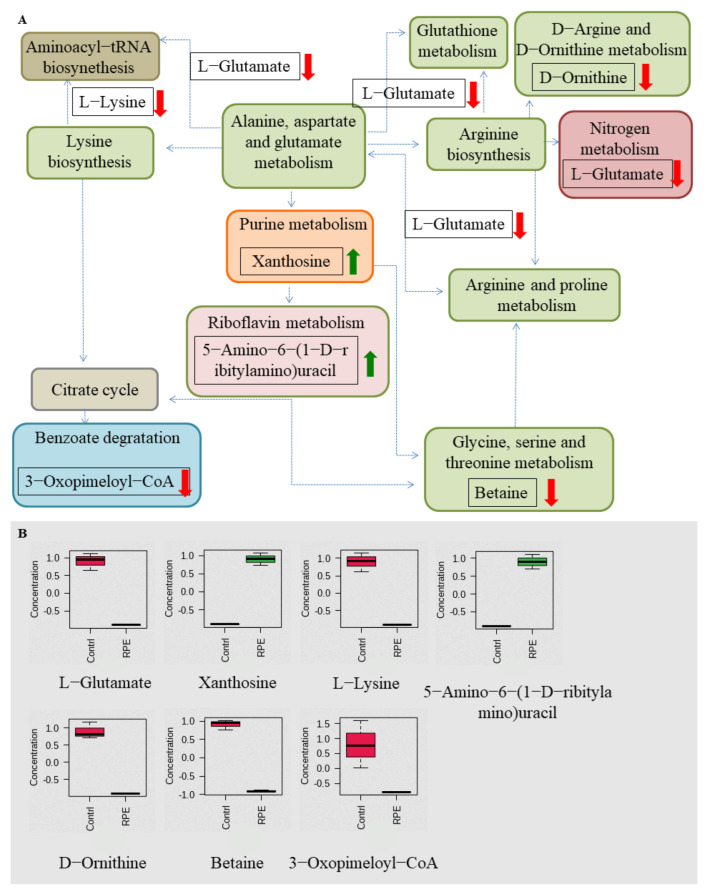
Related metabolic pathways and metabolites with significant changes following RPE treatment in *S. aureus* compared to the untreated group. (**A**) Green and red arrows indicate an increase and decrease abundance of metabolites, respectively. Different colors in the pathway represent different types of metabolism, while the same color represents the same type of metabolism, e.g., green are all amino acid metabolism. (**B**) Box plots for metabolites with significant changes involved in related metabolic pathways.

The metabolic differences of *S. aureus* and MRSA following RPE treatment were investigated relative to untreated controls. After RPE treatment for 24 h, alterations in *S. aureus* and MRSA intracellular metabolites were analyzed. For *S. aureus*, a total of 984 metabolites were identified, and analyzed by ANOVA (*p* < 0.05) highlighting 428 differential metabolites. For MRSA, a total of 2511 metabolites were identified, including 161 differential metabolites (*p* < 0.05, analyzed by ANOVA). Orthogonal partial least-squares discriminant analysis (OPLS-DA) was applied for the recognition of the sample patterns, notably, the OPLS-DA spots (Figure 5) and showed the RPE treatment on *S. aureus* and MRSA group was significantly separated from relevant control groups. Enrichment analysis was carried out according to The Kyoto Encyclopedia of Genes and Genomes (KEGG) to categorize the differential metabolites into related pathways. The results indicated that 12 pathways were enriched after treatment with RPE treated on *S. aureus*. These included aminoacyl-tRNA biosynthesis; lysine biosynthesis; alanine, aspartate, and glutamate metabolism; glutathione metabolism; d-Arginine and d-ornithine metabolism; nitrogen metabolism; purine metabolism; glycine, serine, and threonine metabolism; riboflavin metabolism; benzoate degradation; arginine and proline metabolism; and arginine biosynthesis between the two groups (Figure 6A), these pathways contain metabolites in which xanthosine and 5-Amino-6-(1-d-ribitylamino)uracil are increased and l-Glutamate, l-Lysine, d-Ornithine, Betaine, and 3-Oxopimeloyl-CoA are decreased (Figure 6B).

**Figure 7:**
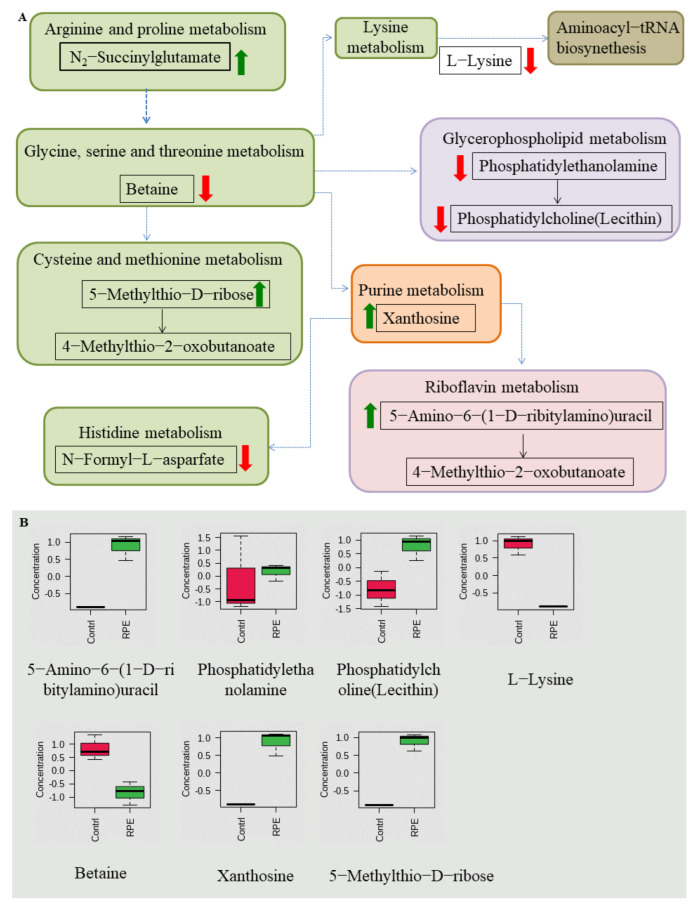
Related metabolic pathways and metabolites with significant changes involved after treatment with RPE treated on MRSA compared to the untreated group. (**A**) Green and red arrows indicate increase and decrease abundance of metabolites, respectively. Different colors in the pathway represent different types of metabolism, while the same color represents the same type of metabolism, e.g., green are all amino acid metabolism. (**B**) Box plots for metabolites with significant changes involved in related metabolic pathways.

Nine pathways were enriched after RPE treated on MRSA, including aminoacyl-tRNA biosynthesis; lysine biosynthesis; glycerophopholipid metabolism; purine metabolism; glycine, serine, and threonine metabolism; riboflavin metabolism; histidine metabolism; arginine and proline metabolism; and cysteine and methionine metabolism between the two groups (Figure 7A), pathways contain metabolites in which N_2_-Succinylglutamate, 5-Methylthio-d-ribose, xanthosine, and 5-Amino-6-(1-d-ribitylamino)uracil are increased, and Betaine, N-Formyl-l-asparfate, l-Lysine, phosphatidylethanolamine, and phosphatidylcholine (Lecithin) are decreased (Figure 7B).

### 2.7. Effect of RPE on the Related Gene Expression of MRSA

**Figure 8:**
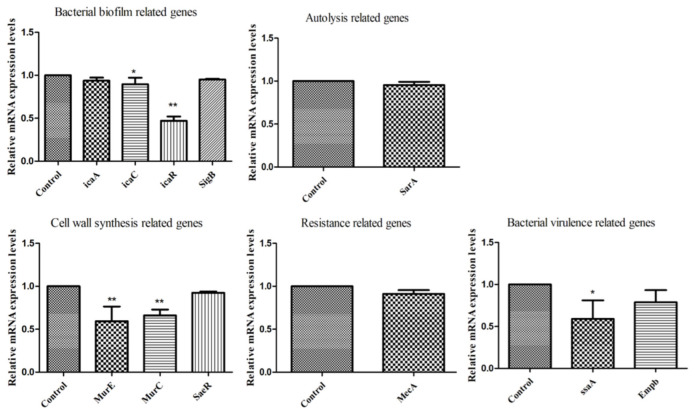
Gene expression of MRSA after exposure to RPE by real-time quantitative reverse transcription PCR (bacterial biofilm-related genes: *icaA*, *icaC*, *icaR*, *SigB*; autolysis-related gene: *SarA*; cell wall synthesis-related genes: *MurE*, *MurC*, *SaeR*; resistance-related gene: *MecA*; bacterial virulence-related genes: *ssaA*, *Empb*). All data are presented as mean values ± SD, and *n* = 3 in each group. * *p* < 0.05, ** *p* < 0.01, vs. the solvent control.

Finally, we analyzed the gene expressions 11 related genes following exposure to RPE in MRSA. As shown in Figure 8 in all genes investigated, expression was downregulated compared to the control group; among them, the expression of icaC and ssaA was significantly downregulated compared to the control group (*p* < 0.05). The expression of icaR, MurE, and MurC were highly significantly downregulated compared to the control group (*p* < 0.01).

## 3. Discussion

This study was carried out to assess the antibacterial activity and mechanism of RPE with a view to counteracting the harmful effects of *S. aureus* and MRSA. First, we quantitatively analyzed 14 main polyphenols in RPE and determined the antibacterial activity of RPE by means of the zone of inhibition, MIC, MBC, and time–growth curve. Subsequently, we investigated the inhibitory effect of RPE on the *S. aureus* and MRSA cell membrane and cell wall disruption by detecting protein and nucleic acid release, AKP release, and by performing SEM analysis. Finally, the metabolomic analysis showed that RPE inhibits *S. aureus* and MRSA by inducing changes in intracellular metabolite concentrations and related metabolic pathways. RPE downregulated genes expression related to bacterial biofilm formation, autolysis, cell wall synthesis, and bacterial virulence in MRSA.

Propolis in China is mainly yellowish brown and dark in color, with a chemical composition that is variable and complex, and dependent on source [28]. For red propolis from China, studies on its constituents and activities are still in the initial stages. It is well known that propolis has been reported for its antibacterial activity, however the mechanism of propolis antibacterial action is not well defined. Most studies suggesting that the antibacterial activity may be due to the synergistic effect of polyphenols contained in propolis [29]. In our study, we selected the 14 main polyphenols of RPE and subjected them to quantitative analysis by LC-QQQ. We found that the red propolis contained high levels of pinobanksin, pinobanksin-3-acetate, and chrysin. Darwish et al. investigated the antibacterial activity of Jordanian propolis of pine origin and oak origin. Three flavonoids: pinobanksin-3-*O*-acetate, pinocembrin, and chrysin were isolated from the propolis of pine origin and studied for antibacterial activity in vitro. Among the results, pinobanksin-3-*O*-acetate and pinocembrin showed antibacterial activity, especially against MRSA, while chrysin was active only against standard *S. aureus* [30]. Kopacz et al. tested morin; sodium salt of morin-5″-sulfonic acid (NaMSA); and new complexes of La (II), Gd (III), and Lu (III) with morin against *Escherichia coli* G (-), *Klebsiella pneumoniae* G (-), and *S. aureus* G (+). The results showed that morin was the most effective against *E. coli* and *S. aureus* [31]. Caffeic acid phenethyl ester showed higher antibacterial activity than 5-chlorogenic acid and caffeic acid against *S. aureus*, *Bacillus subtilis*, and *Pseudomonas aeruginosa* in an in vitro study by Kishimoto et al. [32]. Kharsany et al. studied the antibacterial activity of pinocembrin, galangin, and chrysin, the main compounds in South African propolis—both singularly and in combination—and found that the action of a single compound in propolis was not as effective as the synergistic action of the compounds in inhibiting bacteria [33].

In general, bacteria in the logarithmic growth phase are relatively stable, more resistant, and also more sensitive to external factors [34,35]. For a more accurate determination of the antibacterial activity of RPE against *S. aureus* and MRSA, the bacteria studied were all in the logarithmic growth phase. Gram-positive bacteria have a thick peptidoglycan layer in the cell wall, but cannot prevent polyphenols from entering the cell [36]. The antimicrobial activity of polyphenols depends on its chemical structure and environmental conditions [37]. The antibacterial activity of RPE against *S. aureus* and MRSA was evaluated and showed inhibitory effects, resulting in a MIC of 100 µg/mL for *S. aureus* and a MIC of 50 µg/mL for MRSA (Table 2). The MIC results of *S. aureus* are similar to those Bonvehiet et al. obtained from propolis, which ranged from 0.080 to 0.100 mg/mL [38], and lower than the MIC found by Rahman [39]. To further confirm the antibacterial activity of RPE against *S. aureus* and MRSA, time–kill curves of *S. aureus* and MRSA were plotted. Growth of *S. aureus* and MRSA was almost completely inhibited after 24 h of incubation at the RPE concentrations of 2 × MIC (Figure 2). These results demonstrated the dose dependency of RPE antibacterial effects.

Bacterial cell membranes are selectively permeable. The cell membrane protects the cell by preventing harmful substances from entering the cell, and also allowing substances that nourish the cell to enter [40], thus the integrity of the cell membrane can impact cell survival [15]. Small biomolecules, such as DNA, are permeable to move in the bacterial cell wall, and protein is important for maintaining the nutritional metabolism of the bacterial organism and for immune regulation [41]; in particular, soluble proteins play an important role in regulating cell permeability [42]. Damage to the integrity of the cell membrane can be determined by the release of proteins and nucleic acids [43]. In our study, the OD260 and OD280 of *S. aureus* and MRSA increased after treatment with increasing concentrations of RPE, suggesting that RPE damaged the cell membrane of *S. aureus* and MRSA (Figure 3). These results are in agreement with other reports of the action of antimicrobials on bacterial cells, thus suggesting that mechanism of action of RPE may be due to loss of membrane integrity [44].

The cell walls of bacteria are robust, and used to maintain the normal shape of bacterial cells, maintain normal pressure, and protect cells from the invasion by external substances [45]. AKP is found between the bacterial cell membrane and the cell wall; AKP is released when the cell wall is disrupted and as such can be used to determine the integrity of the bacterial cell wall [46]. AKP leakage from *S. aureus* (Figure 4A) and MRSA (Figure 4B) increased after treatment with increasing concentrations of RPE. SEM was used to study the bacteriostatic mechanism, with SEM results showing that *S. aureus* and MRSA all have a regular oval shape when untreated. However, following RPE treatment, the normal shape of the cells is disrupted and the contents are released (Figure 4C), suggesting that the action of RPE against *S. aureus* and MRSA is via damage to the cell wall—a theory further supported by the leakage of DNA and protein studies that we conducted. These results were in agreement with the conclusions of previous studies [47,48]. 

In addition to the above studies, changes in bacterial metabolism can also cause bacterial death [49], and antibacterial mechanisms can also be investigated by studying changes in the metabolic pathways of bacterial metabolites metabolic pathways [50]. By further studying the changes in metabolites and metabolic pathways following the action of RPE on *S. aureus* and MRSA, we hypothesize that new discoveries can be made regarding the antibacterial mechanism of RPE. In our study, the analysis of the metabolites in intracellular metabolites using a nontargeted metabolomics approach, the OPLS-DA spots (Figure 5) revealed the measurements from *S. aureus* and MRSA treated with RPE were significantly separated from the control group and clustered in defined ranges. In the results of the metabolic pathway analysis of *S. aureus*, a total of 7 metabolites in 12 metabolic pathways showed significant changes (Figure 6). Of these, 5 metabolites decreased in 10 metabolic pathways. The metabolite l-Glutamate decreased in aminoacyl-tRNA biosynthesis; alanine, aspartate, and glutamate metabolism; nitrogen metabolism; glutathione metabolism; arginine and proline metabolism; and arginine biosynthesis. l-Lysine decreased in aminoacyl-tRNA biosynthesis and lysine biosynthesis; d-Ornithine decreased in d-Arginine and d-ornithine metabolism; betaine decreased in glycine, serine, and threonine metabolism; and 3-Oxopimeloyl-CoA decreases benzoate degradation. Two metabolites increased in two metabolic pathways, the metabolite xanthosine is increased in purine metabolism, and 5-Amino-6-(1-d-ribitylamino)uracil is increased in riboflavin metabolism. Of the above metabolic pathways, all lysine biosynthesis; alanine, aspartate, and glutamate metabolism; glutathione metabolism; arginine and proline metabolism; glycine, serine, and threonine metabolism; arginine biosynthesis; and d-arginine and d-ornithine metabolism are involved in amino acid metabolism, indicating that amino acid metabolism plays a very important role in antibacterial activity, which is consistent with Chen’s [22] view. In previous studies, the metabolic pathways enriched by differential metabolites such as arginine and proline metabolism; alanine, aspartate, and glutamate metabolism; aminoacyl-tRNA biosynthesis [22]; purine metabolism [51]; glycine, serine, and threonine metabolism [52]; and nitrogen metabolism [53] were also detected. 

In the results of the metabolic pathway analysis of MRSA, a total of 11 metabolites in the 9 metabolic pathways showed significant changes (Figure 7). Of these, five metabolites decreased in five metabolic pathways. The metabolite l-Lysine was decreased in aminoacyl-tRNA biosynthesis and lysine biosynthesis; betaine is decreased in glycine, serine, and threonine metabolism; n-Formyl-l-asparfate is decreased in histidine metabolism, phosphatidylethanolamine; and phosphatidylcholine (lecithin) is decreased in glycerophopholipid metabolism. Four metabolites increased in four metabolic pathways. The metabolite N2-succinylglutamate increases in arginine and proline metabolism, 5-Methylthio-d-ribose increases in cysteine and methionine metabolism, xanthosine increases in purine metabolism, and 5-Amino-6-(1-d-ribitylamino) uracil increases in riboflavin metabolism. Of the above metabolic pathways, all arginine and proline metabolism; cysteine and methionine metabolism; glycine, serine, and threonine metabolism; lysine biosynthesis; and histidine metabolism are involved in amino acid metabolism, also indicating that amino acid metabolism plays a very important role in antibacterial activity in MRSA. In previous studies, the metabolic pathways enriched by differential metabolites—such as arginine and proline metabolism; purine metabolism [53]; glycine, serine, and threonine metabolism [52]; aminoacyl-tRNA biosynthesis; and cysteine and methionine metabolism [22]—glycerophopholipid metabolism and histidine metabolism [54] were also detected.

We further determined the changes in expression of relevant genes of MRSA following treatment with RPE. A very important factor affecting MRSA resistance is the formation of biofilms; biofilm formation can effectively prevent infection. Biofilm formation is associated with many cell surface and secreted virulence factors [55]. The formation of a bacterial biofilm begins with the attachment of bacteria to an external surface, followed by the formation of a complex membrane structure, a process that critically depends critically on the synthesis of polysaccharide intercellular adhesion (PIA) [56], which is regulated by the ica operon, which contains *icaA*, *icaB*, *icaC*, *icaD*, and *icaR* [57]. In the present study, we detected that the bacterial biofilm-related gene (*icaA*, *icaC*, *icaR*, and *SigB*) expression levels, as shown in Figure 8, were downregulated compared with control group, suggesting that RPE may prevent biofilm formation through the altering the PIA pathway. Bacterial virulence-related genes, including *ssaA* and *Empb*, were downregulated compared with control group. *ssaA* was significantly downregulated (*p* < 0.05), indicating that RPE treatment weakened the secretion of bacterial virulence factors. The autolysis-related gene—*sarA*, also associated with the release of virulence factors and the formation of biofilms [58]—was also downregulated, a result consistent with a previous study [59]. Expression of the cell wall synthesis-related genes, *MurE* and *MurC*, was significantly inhibited compared with control group (*p* < 0.01), which was consistent with AKP leakage and SEM results, suggesting that RPE can kill MRSA by inhibiting and disrupting cell wall synthesis.

## 4. Materials and Methods

### 4.1. Extraction of CRP

CRP samples were collected from Shandong, China, and the samples were extracted with 95% *v*/*v* ethanol as solvent with the ultrasound assisted method [60], and the extracts were concentrated by rotary evaporation (Switzerland Buchi, R-300) to obtain constant weight CRP. The RPE was then dissolved in methanol to obtain a stock solution at a concentration of 50 mg/mL, which was stored at 4 °C

### 4.2. Calibration and Quantification of the Main Polyphenols in RPE

The RPE stock solution was diluted to a concentration of 1 mg/mL with methanol. Fourteen standards—pinobanksin-3-acetate, morin, ferulic acid, caffeic acid, caffeic acid phenethyl ester, pinobanksin, galangin, quercetin, p-coumaric acid, apigenin, kaempferol, chrysin, pinocembrin, and naringenin—were prepared at a concentration of 100 mg/mL in 100 µL of methanol. The standard stock solutions were then mixed in equal parts to maintain a concentration of each standard of 1 mg/mL and were then diluted to the following concentrations in methanol: 100, 200, 400, 600, and 800 µg/mL, a calibration curve was constructed by injecting each standard solution at each concentration level. The RPE and mixed standard solutions of different concentrations were filtered through a 0.22 µm membrane filter into a LC bottle ready to be assayed.

Agilent 6495 Triple Quadrupole LC/MS was applied to quantify the 14 major polyphenols in RPE. Analysis was performed on an Agilent Infinity Lab Poroshell SB-C18 (3.0 × 100 mm, 2.7 µm) with the column temperature set at 35 °C, mobile phase A was 0.1% *v*/*v* formic acid-water and phase B was methanol, with an injection volume of 2 µL and a flow rate of 0.25 mL/min. The gradient elution procedure was as follows: 5–55% B in 1–6 min; 55–95% B in 6–20 min; 95–5% B in 20–21.5 min, post-run time was 5 min. The electrospray ionization source [15] was used for mass spectrometry, and the samples were detected in negative ion mode with the following parameters: gas temp 270 °C, carrier gas (N_2_) flow rate 10 L/min, atomization pressure 25 psi, sheath gas temperature 350 °C, sheath gas flow rate 12 L/min, capillary voltage (Vcap) 4000 V, and nozzle voltage 1500 V.

### 4.3. Bacterial Strains and Growth Conditions

*S. aureus* (ATCC 25923) and MRSA (ATCC 43300) strains were obtained from China Center of Industrial Culture Collection. It is usually acknowledged that the logarithmic phase cells are more sensitive to external stress factors, while stationary phase cells are more resistant [33,34]. The bacteria strains were cultured overnight to the logarithmic phase of growth at 37 °C (approximately 16 h) in Luria-Bertani broth culture medium (AOBOX Biotechnology, Beijing, China). Both inoculums were measured at OD600 and adjusted to the concentration of 5 × 10^7^ CFU/mL using the McFarland standard. Culture purity was examined by streaking each culture on plates of LB agar and nutrient agar for *S. aureus* and MRSA and incubating at 37 °C overnight.

### 4.4. Determination of Antibacterial Activity of RPE

RPE antimicrobial activity was assessed using the agar disk diffusion method [61]. Bacterial cultures of *S. aureus* and MRSA were spread evenly on LB plates with applicator sticks at a concentration of 5 × 10^7^ CFU/mL. Wells of 6 mm in diameter were bored into LB solid agar and each well filled with 100 µL RPE with a concentration of 100 µg/mL or control (methanol) separately. Following incubation overnight at 37 °C the zone of inhibition was measured and reported as diameter in mm.

### 4.5. Determination of Minimal Inhibitory Concentration and Minimum Bactericidal Concentration (MBC)

The MIC and MBC of RPE against *S. aureus* and MRSA were determined following the Clinical and Laboratory Standards Institute 2017 (CLSI 2017) by the gradient dilution assay [52]. RPE was dissolved in methanol and prepared in LB broth medium ranging from 50 µg/mL to 600 µg/mL, methanol was used as a solvent control. After incubation at 37 °C for 24 h, 50 µL of the cultures were spread evenly on LB plates with applicator sticks (5 × 10^7^ CFU/mL) and incubated at 37 °C for 16 h to identify the lowest RPE concentration that inhibited bacterial growth as MIC and the bacterial concentration that showed no bacterial growth as MBC.

### 4.6. Time–Growth Curve Assay

The time–growth analysis of RPE against *S. aureus* and MRSA were performed using previous methods with slight modifications [62], RPE was tested at a concentration of MIC and 2 × MIC was added to logarithmic phase of growth *S. aureus* and MRSA suspensions (5 × 10^7^ CFU/mL), while methanol was used as a solvent control. After incubation at 37 °C for 0, 4, 8, 12, and 24 h, 200 µL of suspension transferred into a 96-well plate and OD600 nm was measured. Growth curves of *S. aureus* and MRSA were constructed by plotting OD600 against time.

### 4.7. Intracellular Protein and Nucleic Acid Leakage Assay

The effects RPE treatment on the leakage of the intracellular contents of *S. aureus* and MRSA was evaluated, according to reported procedures with some modifications [63,64]. RPE was added to an overnight culture of *S. aureus* and MRSA suspensions (5 × 10^7^ CFU/mL), respectively both in logarithmic phase of growth at 37 °C. Tests were performed at final concentrations of RPE of MIC and 2 × MIC, whilst bacterial culture exposed to equivalent methanol concentration as solvent control. Bacterial solutions were incubated at 37 °C for 24 h, then centrifuged at 8000× *g* for 10 min. The optical density of the supernatant was then measured at 260 and 280 nm using an UV spectrophotometer (JING HUA) in order to determine the release of intracellular nucleic acid and protein respectively.

### 4.8. Alkaline Phosphatase (AKP) Release Determination and Scanning Electron Microscopy (SEM)

To examine the mechanisms of action of RPE against cell wall of *S. aureus* and MRSA, AKP release was evaluated according to the instruction of AKP assay kit (Beyotime Biotechnology) [65], SEM studies were carried out as previously reported with some modifications [66]. Logarithmic growth phase cells of *S. aureus* and MRSA (each approximately 5 × 10^7^ CFU/mL) were treated with RPE at the same concentration (100 µg/mL). The control and RPE treated *S. aureus* and MRSA were incubation at 37 °C for 24 h. After incubation, cells were collected by centrifugation for 10 min at 8000× *g* and washed twice with 0.1 M phosphate buffer solution (PBS, PH = 7.0), then were resuspended in 2.5% glutaraldehyde and kept overnight at −4 °C to fix the cells. After centrifugation, the cells were further dehydrated in ethanol with increasing concentrations respectively (35%, 50%, 75%, 90%, and 100% *v*/*v*). The dried samples were observed using Hitachi S-750 scanning electron microscopy (Hitachi Company, Tokyo, Japan).

### 4.9. Metabolomic Analysis

Bacterial metabolites of *S. aureus* and MRSA treated with RPE were determined using metabolomic analysis using an Agilent 6545 LC-QTOF-MS/MS. First, RPE was co-cultured with *S. aureus* and MRSA separately at a concentration of 2 × MIC for 24 h (37 °C) in LB broth medium with an initial concentration of 5 × 10^7^ CFU/mL of bacterial solution, methanol in the bacterial culture broth was used as a solvent control. The culture broth was removed from the incubator, centrifuged at 8000× *g* for 10 min, and the supernatant removed. The remaining pellet was washed with PBS and transferred to a 1.5 mL centrifuge tube and then quenched in liquid nitrogen. Samples were removed from liquid nitrogen and 1 mL of a methanol/acetonitrile/water solvent mixture (2:2:1, *v*/*v*) was added and vortexed, followed by repeated freeze–thawing cycle between liquid nitrogen and a 37 °C water bath three times then the samples were left to stand for 1 h at −20 °C and centrifuged at 13,000 rpm for 15 min at 4 °C. The supernatant was taken and spun dry using a rotary evaporator. 100 µL of water/acetonitrile (1:1, *v*/*v*) was added again and sonicated, and the supernatant was centrifuged through a 0.22 µm into an LC bottle ready for assay.

### 4.10. Real-Time PCR (RT-PCR) Analysis Related Gene Expressions of MRSA

Bacterial biofilm-related genes (*icaA*, *icaC*, *icaR*, *SigB*), autolysis-related gene (*SarA*), cell wall synthesis-related genes (*MurE*, *MurC*, *SaeR*), resistance-related gene (*MecA*), and bacterial virulence-related genes (*ssaA*, *Empb*) were selected for quantitative analysis by RT-PCR. MRSA bacteria was centrifuged after incubation in LB broth medium for 12 h at 37 °C and resuspended in TRIzol regent containing 100 µg/mL lysostaphin. Total RNA was extracted using RNasy mini kit (Qiagen, Düsseldorf, Germany) and then converted to cDNA using PrimeScript RT reagent kit (TaKaRa) according to the manufacturer’s instructions. The RT-PCR reaction using cDNA templates were performed with TaKaRa TB Green™ Premix Ex Taq™ II (Tli RNaseH Plus; RR820A) on QuantStudio1 applied biosystems. The cycle threshold values of all tested genes were normalized using *GAPDH* as reference gene, gene expressions were calculated by 2^(−^^ΔΔ^^CT)^ method [67]. The primers [68] used are listed in Table S1.

### 4.11. Statistical Analysis

A completely randomized design with three replications was performed and the data was expressed as the mean ± standard deviation. One-way analysis of variance (ANOVA) using SPSS20.0 was used to identify any significant differences between the means using a 95% confidence interval (*p <* 0.05).

## 5. Conclusions

On basis of these results, it may be concluded that RPE was rich in pinobanksin, pinobanksin-3-acetate, and chrysin and possessed considerable antibacterial activities against *S. aureus* and MRSA. RPE was found to inhibit *S. aureus* and MRSA by disrupting the cell wall, cell membrane, and induce changes in cell morphology. In addition, metabolomic analysis further revealed differences in bacterial metabolism following RPE treatment in *S. aureus* and MRSA. Finally, RPE downregulated gene expression related to bacterial biofilm formation, autolysis, cell wall synthesis, and bacterial virulence of MRSA. Thus, our study provides novel mechanistic insight to understand RPE action against *S. aureus* and MRSA. The findings of this study suggest RPE may be a promising therapeutic agent for use against *S. aureus* and MRSA.

## Figures and Tables

**Table 1:** Quantitative results for 14 polyphenols in RPE.

Compound Name	Retention Time	Precursor (*m/z*)	Product Ions (*m/z*)	CE	RPE (mg/g)
Pinobanksin-3-acetate	7.813	313.1	253.1	15	53.79
			271.0	15	
Morin	6.609	301	151	16	1.04
			125	16	
Ferulic acid	5.487	192.9	178.1	15	2.82
			134.2	10	
Caffeic acid	4.798	179	135	10	8.75
			107.2	20	
Caffeic acid phenethyl ester	7.995	283.1	179	15	14.39
			135.1	25	
Pinobanksin	6.819	271.1	253	15	194.05
			125	20	
Galangin	8.268	269.1	213.1	20	25.33
			171	25	
Quercetin	6.609	301	179	18	1.01
			151	22	
P-coumaric acid	5.384	163.1	116.8	30	8.75
			119	10	
Apigenin	7.242	269	151.1	20	14.39
			117.1	40	
Kaempferol	7.114	285	257	20	1.74
			93	35	
Chrysin	8.188	253.1	143	24	44.51
			63	32	
Pinocembrin	7.811	255.1	213	15	18.66
			151.2	20	
Naringenin	6.685	271.1	151	10	0.67
			119	25	

CE, collision energy.

**Table 2:** Antibacterial activity of RPE in terms of MIC and MBC against *S. aureus* and MRSA.

Sample	*S. aureus* ATCC25923		MRSA ATCC43300	
	MIC (µg/mL)	MBC (µg/mL)	MIC (µg/mL)	MBC (µg/mL)
RPE	100	300	50	200

## Supplementary Materials

The following are available online at https://www.mdpi.com/article/10.3390/molecules27051693/s1. Figure S1: MS/MS spectra of 14 polyphenol; Table S1: Primer sequence information for MRSA-related genes.

## Sample Availability

Chinese red bee propolis samples, 14 polyphenol standardsare available from the authors.
