# Supplementary material for: Antibacterial Activity of Chinese Red Propolis against Staphylococcus aureus and MRSA
Source: Molecules. 2022 Mar 4;27(5):1693. doi: 10.3390/molecules27051693 (PMC8911571; doi:10.3390/molecules27051693)
Supplement: Supplementary file 1 [file molecules-27-01693-s001.zip › molecules-1546882-supplementary.pdf]

Supplementary Table S1. Primer sequence information for MRSA-related genes.

ID	Primer name	Gene sequence (5'to3')
1	agrA	F:TGATAATCCTTATGAGGTGCTT<br>R:CACTGTGACTCGTAACGAAAA
2	icaA	F:CTTGCTGGCGCAGTCAATAC<br>R:GTAGCCAACGTCGACAACTG
3	icaB	F:CCTGTAAGCACACTGGATGG<br>R:TCGCTTTTCTTACACGGTGA
4	icaC	F:TGCGTTAGCAAATGGAGACT<br>R:TGCGTGCAAATACCCAAGAT
5	icaD	F:TGGGCATTTTCGCGATTATCA<br>R:ACGATTCTCTTCCTTTCTGCCA
6	icaR	F:TGCTGTTTCTTGAAAGTTGGTATT<br>R:TTTACTTTCTTCCACTGCTCCA
7	SigB	F:TCGCGAACGAGAAATCATACAA<br>R:ACCGATACGCTCACCTGTCTCT
8	SarA	F:GTAATGAGCATGATGAAAGAACTGT<br>R:CGTTGTTTGCTTCAGTGATTCG
9	MecA	F:TCCAGGAATGCAGAAAGACC<br>R:TTGGAACGATGCCTATCTCA
10	Empbp	F:TCGCGTGAATGTAGCAACAAA<br>R:ACTTCTGGGCCTTTAGCAAC
11	ssaA	F:GCTACAGCAGGACTTGCCACTA<br>R:TTATTTTGTTCTGCGGCTTGTG
12	MurC	F:TCGGATGACATTTATGCTCAAAA<br>R:ACCATCCACATACACATCAAAAGC
13	MurE	F:TTAGCATCAAAAGGAAGAGAACCA<br>R:ATCATCTCGATGTGGCACCTTA
14	saeR	F:GCCAAAACACAAGAACATGATACC<br>R:CCCTTGGACTAAATGGTTTTTTGA
15	16S(atcc43300)	F:CGCAATGGGCGAAAGC<br>R:TACGATCCGAAGACCTTCATCA

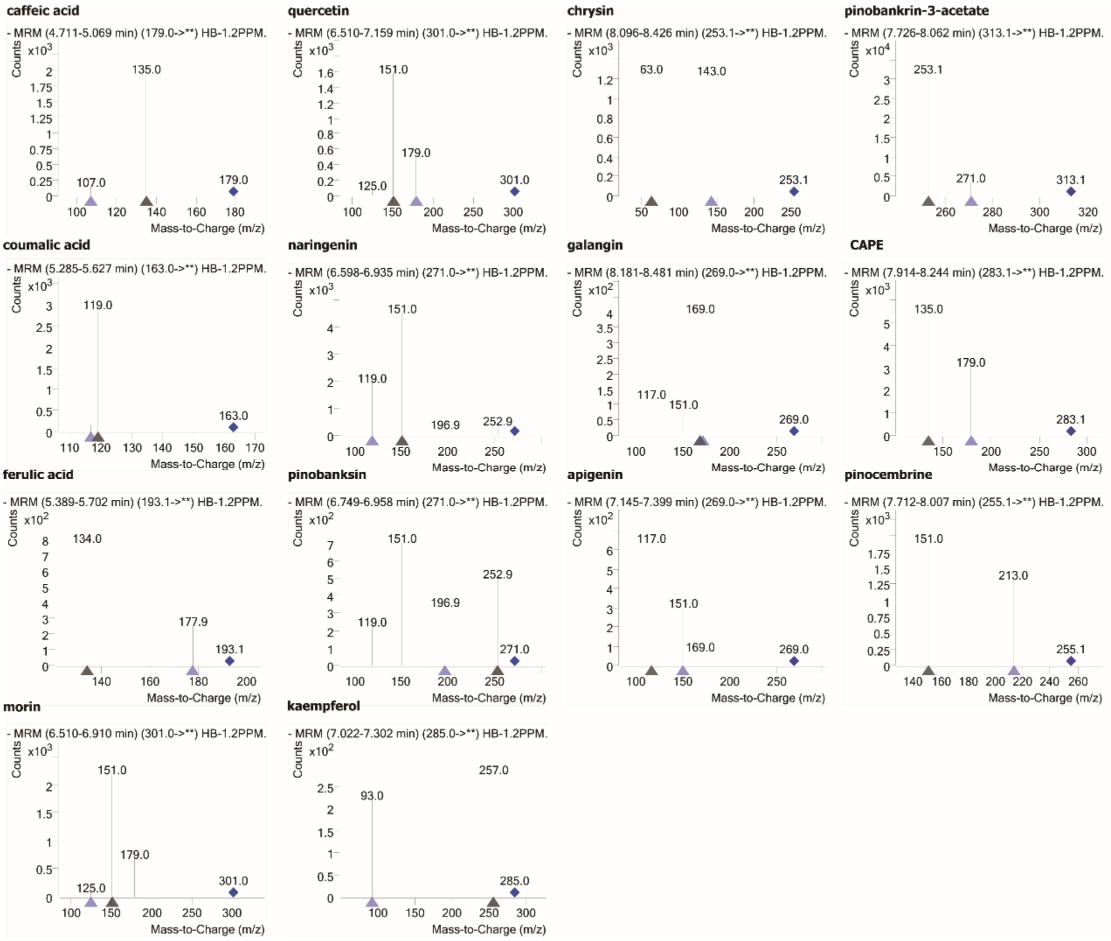

Supplement Figure S1. MS/MS spectra of 14 polyphenols.
